# Lanolin as an Excipient

**Published:** 1907-10

**Authors:** 


### Lanolin as an Excipient.

Lanolin is a good excipient for pills where there is an object in having the drug contained to reach the intestines unchanged by its passage through the stomach. It is not dissolved by the gastric juice but is broken up by the fat splitting process of intestinal digestion.

